# Multiplex PCR Targeted Amplicon Sequencing (MTA-Seq): Simple, Flexible, and Versatile SNP Genotyping by Highly Multiplexed PCR Amplicon Sequencing

**DOI:** 10.3389/fpls.2018.00201

**Published:** 2018-03-23

**Authors:** Yoshihiko Onda, Kotaro Takahagi, Minami Shimizu, Komaki Inoue, Keiichi Mochida

**Affiliations:** ^1^Cellulose Production Research Team, RIKEN Center for Sustainable Resource Science, Yokohama, Japan; ^2^Kihara Institute for Biological Research, Yokohama City University, Yokohama, Japan; ^3^Graduate School of Nanobioscience, Yokohama City University, Yokohama, Japan; ^4^Institute of Plant Science and Resource, Okayama University, Okayama, Japan

**Keywords:** marker panel, SNP, genotyping, amplicon sequence, natural accession, *Brachypodium distachyon*

## Abstract

Next-generation sequencing (NGS) technologies have enabled genome re-sequencing for exploring genome-wide polymorphisms among individuals, as well as targeted re-sequencing for the rapid and simultaneous detection of polymorphisms in genes associated with various biological functions. Therefore, a simple and robust method for targeted re-sequencing should facilitate genotyping in a wide range of biological fields. In this study, we developed a simple, custom, targeted re-sequencing method, designated “multiplex PCR targeted amplicon sequencing (MTA-seq),” and applied it to the genotyping of the model grass *Brachypodium distachyon*. To assess the practical usability of MTA-seq, we applied it to the genotyping of genome-wide single-nucleotide polymorphisms (SNPs) identified in natural accessions (Bd1-1, Bd3-1, Bd21-3, Bd30-1, Koz-1, Koz-3, and Koz-4) by comparing the re-sequencing data with that of reference accession Bd21. Examination of SNP-genotyping accuracy in 443 amplicons from eight parental accessions and an F_1_ progeny derived by crossing of Bd21 and Bd3-1 revealed that ~95% of the SNPs were correctly called. The assessment suggested that the method provided an efficient framework for accurate and robust SNP genotyping. The method described here enables easy design of custom target SNP-marker panels in various organisms, facilitating a wide range of high-throughput genetic applications, such as genetic mapping, population analysis, molecular breeding, and genomic diagnostics.

## Introduction

Recent advances in high-throughput sequencing technologies have accelerated research and developments in the field of genomics (Metzker, 2010; Schneeberger, 2014). The dramatic increase in sequenced genomes provides substantial benefits to genomics-based approaches. Currently (as of April 12, 2017), the whole-genome data for 755 animal and 247 plant species are publicly available (http://www.ncbi.nlm.nih.gov/genome/browse/). These datasets provide valuable information for developing genetic tools to discover useful genes for applications in molecular breeding and diagnostics.

In forward-genetics approaches, genotyping at a genome, as well as a population scale, is the primary step to elucidate associations between genetic polymorphisms and traits of interest, which is essential to perform genetic mapping in quantitative trait locus (QTL) analyses and genome-wide association studies (Schneeberger and Weigel, 2011; Schneeberger, 2014). Single-nucleotide polymorphisms (SNPs) are the most frequently observed mutations and are useful as genetic markers. Because of their co-dominant segregation patterns, SNPs are often more informative than the dominant markers. A previous review showed that pooled next-generation sequencing (NGS) with barcoding is an efficient approach to genotype selected candidate genes for several hundreds of individuals (Marroni et al., 2012). This approach has been applied with excellent results in plants, such as wheat, rice, and poplar (Kharabian-Masouleh et al., 2011; Marroni et al., 2011; Tsai et al., 2011). Recently, several time- and cost-effective methods for SNP genotyping through high-throughput sequencing, such as restriction-site-associated DNA sequencing and genotyping-by-sequencing, have been widely used to elucidate genetic diversity (Davey et al., 2011; Onda and Mochida, 2016). However, because these methods do not target specific polymorphisms, the frequency of commonly called SNP sites is expected to decline along with increasing population size (Marchini and Howie, 2010; Rutkoski et al., 2013; Fu, 2014; Fu et al., 2016), and, therefore, they might not be suitable for genotyping panels.

The sequencing of multiplex PCR-based amplicons is an ideal approach to assaying a polymorphic panel of several hundreds of target SNP markers at the population scale and using a wide range of genetic applications, such as phylogenetic analysis, population structure analysis, and QTL mapping (Brachi et al., 2011; Andrews et al., 2016). Currently, commercial kits for amplicon sequencing are available, including the TruSeq Custom Amplicon developed by Illumina (Csernák et al., 2016) and the Ion AmpliSeq Custom DNA Panels marketed by Thermo Fisher Scientific (Millat et al., 2014; Glotov et al., 2015). Moreover, targeted amplicon sequencing effectively allows genotyping of small amounts of DNA derived from samples, such as dried herbarium and fossil specimens (Gugerli et al., 2005; Délye et al., 2013; Beck and Semple, 2015). Therefore, SNP marker panels optimized for multiplexed amplicon sequencing are useful in various research fields, including evolutionary, ecological, agricultural, and population genetics (Savolainen et al., 2013; Tiffin and Ross-Ibarra, 2014; Kumar et al., 2015).

*Brachypodium distachyon* is an annual grass species that belongs to the Pooideae subfamily and is phylogenetically related to economically important temperate cereals, such as wheat and barley (Draper et al., 2001; Bevan et al., 2010; Mur et al., 2011). *B. distachyon* is proposed as a model plant for the study of biological phenomena in temperate cereals and biofuel crops due to its tractable features, such as small body size, simple growth requirements, self-fertility, a short life cycle, and a small, diploid genome (Bevan et al., 2010; Girin et al., 2014; Kellogg, 2015). It is expected that the genetic diversity found in natural accessions of *B. distachyon* is useful for examining genetic polymorphisms associated with traits involved in adaptation to local environments (Filiz et al., 2009; Vogel et al., 2009; Mur et al., 2011; Gordon et al., 2014). To characterize the natural and genotypic diversity in Turkish populations of *B. distachyon*, Vogel et al. (2009) developed 398 simple sequence repeat markers to determine phylogenetic relations among 187 natural accessions, which indicated considerable genetic diversity. Eight independent inbred lines developed from accessions from Kozluk (“Koz accessions”) showed remarkable phenotypic differences, such as the existence or non-existence of hairs on the lemma (Vogel et al., 2009) and differential disease responses to infection with barley stripe mosaic virus (BSMV) (Cui et al., 2012), although the Koz accessions showed modest genetic diversity and were on the same branch of the phylogenetic tree based on the 187 natural accessions (Vogel et al., 2009). Additionally, *Bsr1*, the BSMV-resistance gene in *B. distachyon*, was mapped using 768 SNP markers designed for the Illumina GoldenGate assay (discontinued by Illumina in 2014) (Huo et al., 2011). A genome-scale SNP panel together with a high-throughput and cost-effective genotyping method without specific platform dependencies would allow gathering polymorphic data from natural accessions and accelerate the mining of genes useful for molecular breeding of related crops, such as wheat and barley.

**Figure 1:**
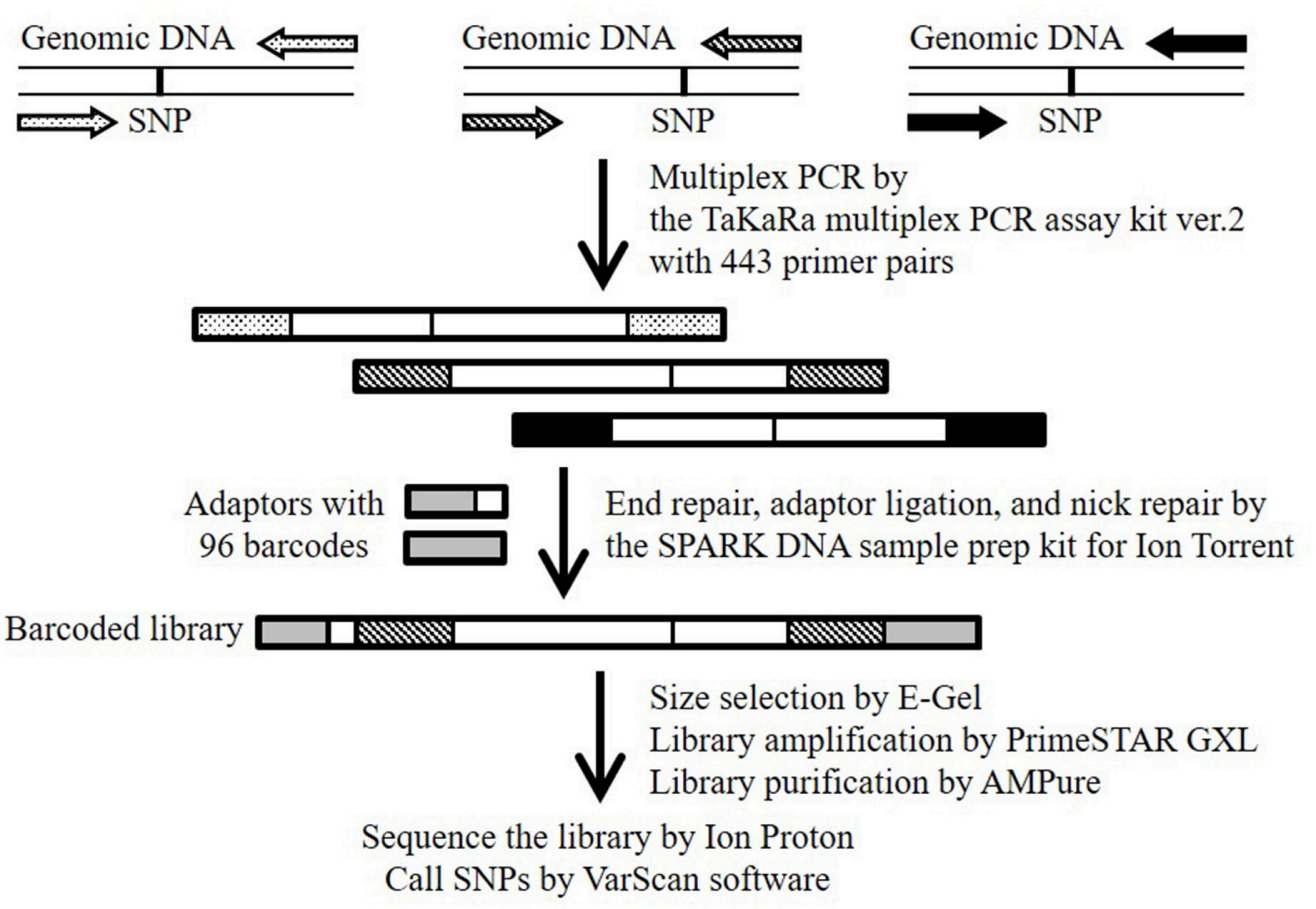
Schematic overview of the MTA-seq workflow used in this study. Primer pairs (443) were designed to generate amplicons harboring one known SNP. Highly multiplexed PCR was conducted with the TaKaRa multiplex PCR amplification kit version 2. Amplicons were end-repaired, adapter-ligated, and nick-repaired with the SPARK DNA sample prep kit for Ion Torrent. To identify individual samples, the Ion Xpress Barcode Adapters 1-96 kit was adopted to add index adapters. The barcoded library was sequenced on an Ion Proton sequencer. SNPs were called by using the “mpileup2cns” command of the VarScan software.

In this study, we report a useful SNP-genotyping method that we termed “multiplex PCR targeted amplicon sequencing (MTA-seq),” which enabled us to genotype >400 SNPs simultaneously (Figure 1). We used the method for high-throughput genotyping of 443 SNPs in eight natural accessions (Bd21, Bd1-1, Bd3-1, Bd21-3, Bd30-1, Koz-1, Koz-3, and Koz-4) of *B. distachyon*, as well as an F_1_ progeny derived by crossing of Bd21 and Bd3-1. We assessed the accuracy and reliability of our method based on genotyping data for homozygous and heterozygous alleles.

## Materials and methods

### Plant materials, DNA purification, and whole-genome re-sequencing

Dry seeds of diploid accessions of *B. distachyon*, Bd21, Bd1-1, Bd3-1, Bd21-3, Bd30-1, Koz-1, Koz-3, and Koz-4, were originally provided by the National Plant Germplasm System of USDA-ARS and were kindly gifted by Dr. David Garvin and Dr. John Vogel. Plants were grown as described previously (Onda et al., 2015). Genomic DNA was extracted from leaves flash-frozen with liquid nitrogen using the DNeasy plant mini kit (Qiagen K.K, Tokyo, Japan). Quality and quantity of the extracted DNA were assessed with a NanoDrop 3300 fluorospectrometer (Thermo Fisher Scientific K. K., Yokohama, Japan). Whole-genome re-sequencing of Bd21, Koz-1, and Koz-4 was conducted with the Ion Proton System (Life Technologies Japan Ltd., Tokyo, Japan). Briefly, DNA libraries for the Ion Proton System were constructed with an Ion Xpress Plus fragment library kit (Life Technologies Japan) and were prepared using the Ion OneTouch 2 System (Life Technologies Japan) for semiconductor sequencing according to manufacturer instructions. The dataset of the Ion Proton System reads has been submitted to the DDBJ Sequence Read Archive under accession numbers DRR092702, DRR092703, and DRR092704 for Bd21, Koz-1, and Koz-4, respectively.

### Production of F_1_ progeny by artificial crossing

Artificial crossing was performed according to the Garvin Lab method (https://www.ars.usda.gov/ARSUserFiles/1931/BrachypodiumCrossing.pdf) and the Vogel Lab method (http://1ofdmq2n8tc36m6i46scovo2e.wpengine.netdna-cdn.com/wp-content/uploads/2015/05/Vogel-lab-Crossing-Brachypodium-2-3-2010.pdf). Anthers were pulled out from the female parent by inserting tweezers into the palea to prevent self-fertility, and the lemma was pushed back to close the floret until pollination. After emasculation, all flowers, except the one targeted for crossing, were removed. Next, flowers with very mature anthers were selected. Appropriate anthers with a yellowish-white color were selected, transferred onto a glass slide, and incubated at 37°C for 5 min to dehisce efficiently. The dehisced anthers were picked up with tweezers and stacked into a female floret to apply the pollen to the stigma. After several days, a developing endosperm was observed if successfully pollinated.

### Genome-wide SNP discovery

Public whole-genome sequence data of natural accessions Bd1-1 (SRS190935), Bd3-1 (SRS290395), Bd21-3 (SRP010886), Bd30-1 (SRS190910), BdTR12c (SRS190847), and Koz-3 (SRS190848) described previously (Gordon et al., 2014) were retrieved from the DDBJ Sequence Read Archive (Table S1). The sequence reads were trimmed using Trimmomatic (version 0.32) (Bolger et al., 2014) with the following settings: -threads 8, -phred33, LEADING: 20, TRAILING: 20, SLIDINGWINDOW:4:15, MINLEN: 36. Because >90% of the sequence data of SRR192296 and SRR400631 were removed by Trimmomatic, these accessions were excluded from subsequent analyses (Table S1). The trimmed reads were mapped to the Bd21 reference genome downloaded from Phytozome (Bdistachyon_192_v1.2, https://phytozome.jgi.doe.gov/pz/portal.html#!bulk?org=Org_Bdistachyon) using the BWA-MEM (v0.7.12) (Li and Durbin, 2009) algorithm, and our whole-genome re-sequencing reads for Koz-1 and Koz-4 were mapped to the Bd21 reference genome using the Torrent Mapping Alignment Program, which is part of the Torrent Suite Software on the Torrent Server (Thermo Fisher Scientific K. K.). Uniquely mapped and properly paired reads (only paired-end reads) with mapping quality ≥10 were extracted using the “view” command in SAMtools (v0.1.19) (Li et al., 2009) and original Perl scripts. Possible duplicated reads were omitted using the SAMtools “rmdup” command. Plural mapping data of the same accession were merged using the SAMtools “merge” command. For SNP calling, we used freebayes (v0.9.21-15-g8a06a0b) (Garrison and Marth, 2012), glfMultiples (v2010-06-16) (The 1000 Genomes Project Consortium, 2010), SAMtools (v0.1.19) (Li et al., 2009), HaplotypeCaller in GATK (v3.3-0) (McKenna et al., 2010), and UnifiedGenotyper in GATK (v3.3-0) (McKenna et al., 2010; DePristo et al., 2011) with default settings to comprehensively identify probable SNPs. A set of SNPs having a read depth of ≥5 and a mapping quality of ≥20 in all SNP call data according to the five different tools were extracted as candidate genotyping markers. Because these genotyping markers were not located at even intervals on the Bd21 reference genome, additional SNPs were sought by using original Perl scripts. SNPs having a read depth of ≥5 and ≥95% different nucleotides as compared with the Bd21 reference genome in all accessions were added to the candidate SNPs of genotyping markers.

### SNP data purification using whole-genome re-sequencing data of Bd21

To reduce unreliable SNPs in the candidate SNP set, we mapped our Bd21 whole-genome re-sequencing reads (DRR092702) to the Bd21 reference genome (Bdistachyon_192_v1.2) with the Torrent Mapping Alignment Program on the Torrent Server (Thermo Fisher Scientific K. K.). Unreliable SNPs having >5% of different nucleotides between our Bd21 reads and the reference genome were removed from the candidate SNP dataset.

### Primer design

Primer3 (v2.2.3) (Koressaar and Remm, 2007; Untergasser et al., 2012) was used to design primer pairs, each of which may be used to amplify a 150–200-bp amplicon that contains one SNP loci. Some options of Primer3 were modified to improve primer specificity; these were as follows: the optimum size of primer was set to 25 bases, ranging from 18 to 32 bases. The optimum melting temperature was set to 65°C, ranging from 60 to 72°C. The range of GC content was set from 45 to 65%. To design primers for the identical genomic regions across the all *B. distachyon* accessions analyzed, we excluded genomic regions, including SNPs, discovered by at least one of the five SNP callers from the input genomic sequences for the primer design.

### Preparation of amplicon libraries, sequencing, and SNP calling

The workflow of the MTA-seq method is shown in Figure 1. Multiplex PCR was performed with the Multiplex PCR assay kit version 2 (TaKaRa, Kusatsu, Japan) according to manufacturer instruction, with modifications. Briefly, 20 ng of genomic DNA (5 ng/μL) was used as a template for multiplex PCR using a Veriti Thermal Cycler (Thermo Fisher Scientific K.K.) and the 443 primer pairs, with 100 μM of each primer used (Table S2). The thermo-cycling conditions were 94°C for 1 min, 30 cycles of 94°C for 30 s and 60°C for 4 min, and a final extension at 72°C for 10 min. The multiplex PCR products were purified using the Agencourt AMPure XP system (Beckman Coulter, Brea, CA, USA). For library preparation, we used the SPARK DNA sample prep kit for Ion Torrent (Enzymatics, Beverly, MA, USA) with an Ion Xpress Barcode Adapters 1–96 kit (Thermo Fisher Scientific K.K.), and size selection was performed using E-Gel SizeSelect (Thermo Fisher Scientific K.K.) according to manufacturer instructions. The SPARK DNA sample prep kit is also available for the Illumina platform. Finally, the library was amplified using PrimeSTAR GXL DNA polymerase (TaKaRa) with the custom forward primer (5′-ATCTCATCCCTGCGTGTCTCC-3′) and reverse primer (5′-TCCGCTTTCCTCTCTATGGGC-3′). The thermo-cycling conditions were 95°C for 5 min, and six cycles of denaturation at 95°C for 15 s, annealing at 58°C for 15 s, and extension at 70°C for 1 min. Amplified products were purified with the Agencourt AMPure XP system and quality checked with the High Sensitivity DNA kit (Agilent Technologies, Santa Clara, CA, USA). Emulsion PCR and template-positive ion-sphere particle enrichment were performed using the Ion OneTouch 2 system and Ion OneTouch ES system, respectively, with the Ion PI Hi-Q OT2 200 kit (Life Technologies Japan). The enriched ion-sphere particles were sequenced with the Ion Proton System with Ion PI Chip using the Ion PI Hi-Q Sequencing 200 kit (Life Technologies Japan). Sequence data were downloaded from the Torrent Server to our in-house server and mapped to the Bd21 reference genome with the Torrent Mapping Alignment Program (Thermo Fisher Scientific). SNPs were called by using the “mpileup2cns” command (http://varscan.sourceforge.net/using-varscan.html#v2.3_mpileup2cns) in the VarScan software (v2.3.7) (Koboldt et al., 2009) using default settings. We verified the results of our SNP genotyping of Bd3-1 and Bd21-3 by whole-genome re-sequencing using the Ion Proton System (the reads has been submitted to the DDBJ Sequence Read Archive under accession numbers DRR124340 and DRR124339, respectively).

### SNP call simulation by random sampling with decreasing read number

The total reads obtained for each accession in this study were randomly sampled to generate 0.01, 0.02, 0.04, 0.07, 0.1, 0.15, 0.2, 0.4, 0.7, 1, 2, and 3 million-read datasets. SNPs were called from the random sampling data with the “mpileup2cns” command of VarScan (v2.3.7) (Koboldt et al., 2009). We performed five independent simulations and calculated the average and standard deviation of the results.

## Results

### Development of a set of even-spaced SNP markers in *B. distachyon*

**Figure 2:**
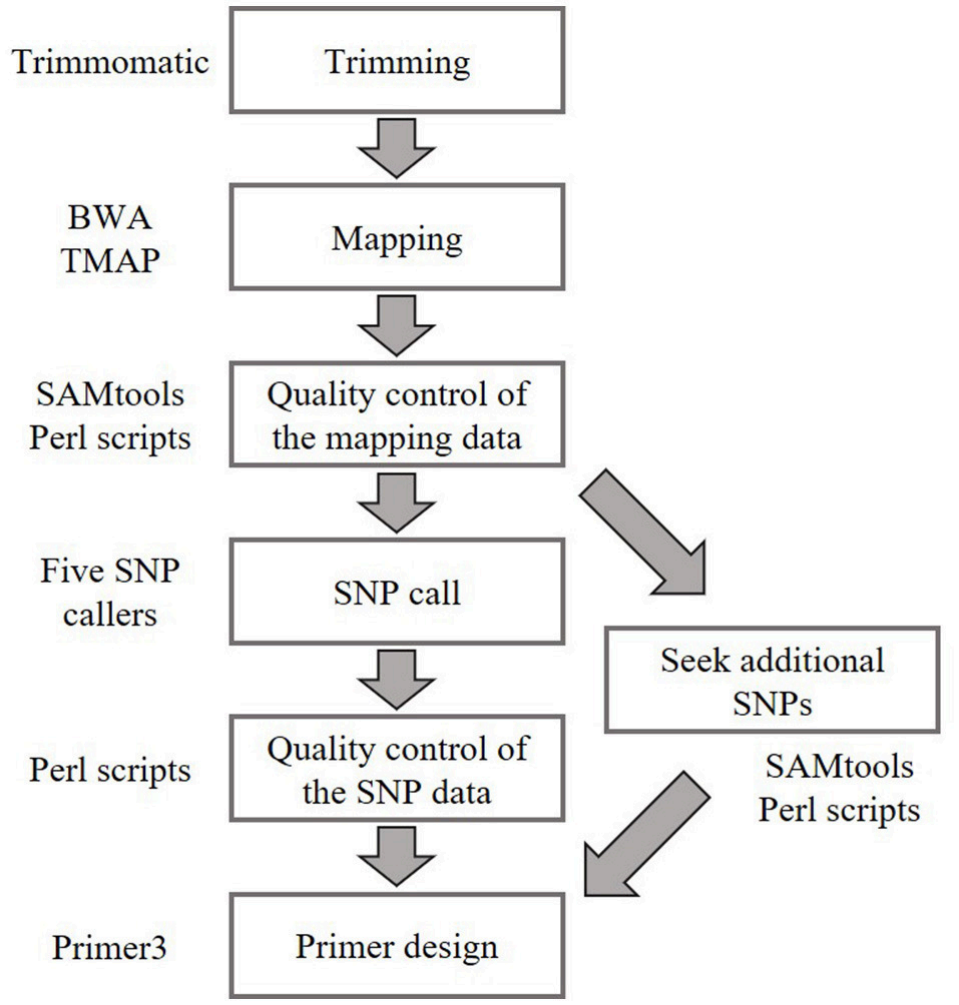
Bioinformatics workflow for SNP identification and primer design in this study. Whole-genome sequence data of natural accessions Bd1-1 (SRS190935), Bd3-1 (SRS290395), Bd21-3 (SRP010886), Bd30-1 (SRS190910), BdTR12c (SRS190847), Koz-3 (SRS190848), Koz-1 (DRR092703), and Koz-4 (DRR092704) were used to identify biallelic SNPs in comparison with Bd21 (The International Brachypodium Initiative, 2010; Gordon et al., 2014).

**Figure 3:**
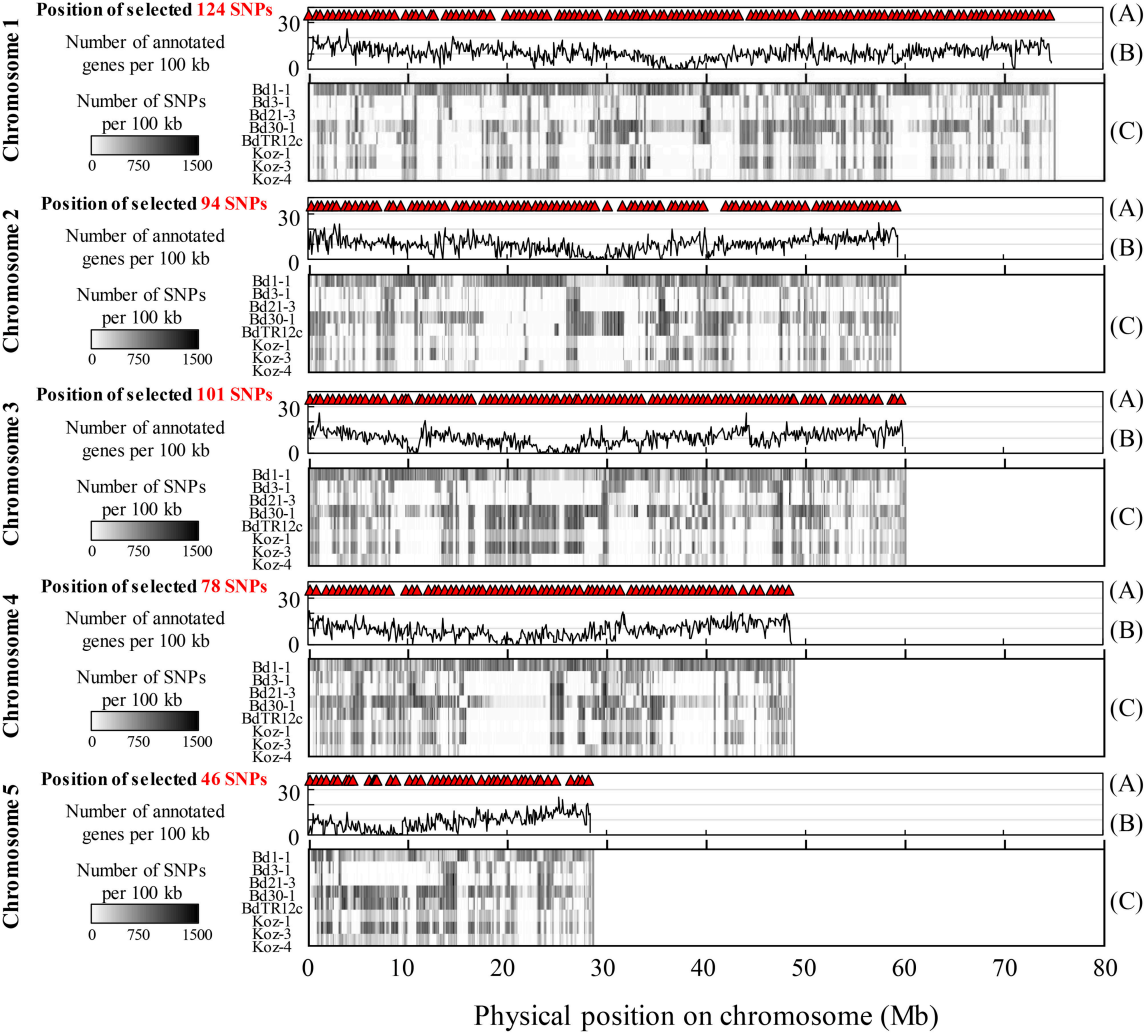
Overview of identified and selected SNPs on each chromosome among the eight natural accessions of *B. distachyon*. **(A)** Positions of the selected SNP markers for MTA-seq. **(B)** Distribution of genes annotated in the Bd21 genome. **(C)** Density of SNPs identified in this study in natural accessions Bd1-1 (SRS190935), Bd3-1 (SRS290395), Bd21-3 (SRP010886), Bd30-1 (SRS190910), BdTR12c (SRS190847), Koz-1 (DRR092703), Koz-3 (SRS190848), and Koz-4 (DRR092704) in comparison with the reference accession Bd21 (Bdistachyon_192_v1.2).

We identified SNPs between the reference accession Bd21 and eight natural accessions (Bd1-1, Bd3-1, Bd21-3, Bd30-1, BdTR12c, Koz-1, Koz-3, and Koz-4) of *B. distachyon*, from which we selected a set of even-spaced SNP markers covering the *B. distachyon* genome as outlined in Figure 2. We performed whole-genome re-sequencing of Koz-1 and Koz-4, which showed remarkable phenotypic differences as compared to Koz-3, with ~21.3 × and ~18.3 × coverages, respectively. For the other six accessions, we retrieved whole-genome sequencing datasets from the DDBJ Sequence Read Archive. By mapping the reads of all eight accessions to the Bd21 genome, we identified between 368,211 and 1,406,967 SNPs between Bd21 and the other accessions (Table 1 and Data Sheets 1–8). The number of SNPs was remarkably higher in Bd1-1 and Bd30-1 than in the other accessions (Table 1 and Figure 3). The genetic diversity of Bd1-1 and Bd30-1 was consistent with their phylogenetic relationships reported previously (Vogel et al., 2009; Gordon et al., 2014), whereas Koz-1 and Koz-4 were rather less divergent (Table 1 and Figure 3). As expected, Bd21-3 showed the fewest SNPs, because Bd21-3 and Bd21 were derived from the same source accession (PI 254867) (Garvin et al., 2008; Vogel and Hill, 2008). We then selected 443 biallelic SNPs distributed almost evenly over the five *B. distachyon* chromosomes. Each of the biallelic SNPs showed a common nucleotide in each of the eight accessions and a polymorphic nucleotide in Bd21. As a result, we selected 124, 94, 101, 78, and 46 SNP markers on chromosomes 1, 2, 3, 4, and 5, respectively. The average distance between adjacent SNPs was ~600 kb on each chromosome (Figure S1), indicating that the two closest markers sandwiched 58.5 ± 21.6 genes annotated in the Bd21 genome on average.

**Table 1:** SNP densities in the accessions used in this study.

**Accession**	**Chromosome 1 74.8 Mb**		**Chromosome 2 59.3Mb**		**Chromosome 3 59.9Mb**		**Chromosome 4 48.6 Mb**		**Chromosome 5 28.4Mb**		**Total 271.0Mb**	
	**Number of SNPs**	**Number of SNPs per 100 kb**	**Number of SNPs**	**Number of SNPs per 100 kb**	**Number of SNPs**	**Number of SNPs per 100 kb**	**Number of SNPs**	**Number of SNPs per 100 kb**	**Number of SNPs**	**Number of SNPs per 100 kb**	**Number of SNPs**	**Number of SNPs per 100 kb**
Bd1-1 (SRS190935)	395,468	528.7	306,400	516.7	307,482	513.3	276,819	569.6	120,798	425.3	1,406,967	519.2
Bd3-1 (SRS290395)	113,893	152.3	87,941	148.3	120,228	200.7	100,577	206.9	49,846	175.5	472,485	174.3
Bd21-3 (SRP010886)	78,231	104.6	67,035	113.0	78,359	130.8	82,555	169.9	40,159	141.4	346,339	127.8
Bd30-1 (SRS190910)	289,419	386.9	242,369	408.7	285,019	475.8	211,074	434.3	149,478	526.3	1,177,359	434.4
BdTR12c (SRS190847)	177,167	236.9	159,215	268.5	182,704	305.0	140,338	288.8	105,451	371.3	764,875	282.2
Koz-1 (DRR092703)	100,001	133.7	75,572	127.4	99,368	165.9	73,219	150.7	54,269	191.1	402,429	148.5
Koz-3 (SRS190848)	174,511	233.3	130,872	220.7	183,222	305.9	129,824	267.1	109,616	386.0	728,045	268.7
Koz-4 (DRR092704)	91,901	122.9	76,128	128.4	86,884	145.0	61,946	127.5	51,352	180.8	368,211	135.9

### Simultaneous genotyping of amplicons

To assess the feasibility of our MTA-seq method for SNP genotyping, we designed PCR primers (Table S2 and Figure 2) targeting each SNP and assessed their accuracy rate in genotyping. For this assessment, we used eight *B. distachyon* accessions, Bd21, Bd1-1, Bd3-1, Bd21-3, Bd30-1, Koz-1, Koz-3, and Koz-4, as well as an F_1_ hybrid derived from crossing of Bd21 and Bd3-1 to assess the genotyping accuracy of MTA-seq for heterozygous alleles. We performed MTA-seq using an Ion Torrent semiconductor sequencer (Ion Proton) and obtained 5.5 million to 7.5 million reads for each accession (Table 2). Among the total reads obtained, >99.5% of the total reads were mapped onto the Bd21 reference genome, and 2.1 million to 3.6 million reads (39.1–50.9% of obtained reads) were mapped onto the genomic regions of the 443 SNPs in each sample (Table 2). The accuracy rate of SNP calling by MTA-seq for each accession was between 95.3 and 97.5% (Table 2), which was also in agreement with our whole-genome re-sequencing data of Bd3-1 and Bd21-3, suggesting that MTA-seq is a feasible method for generating amplicons covering >400 SNP markers in one tube, as well as for the simultaneous genotyping of these amplicons using high-throughput sequencers. Over 90% of the SNP markers (400 of 443 SNPs, indicated by asterisks in Table S3) were adequately amplified and accurately genotyped in all of the nine tested samples.

**Table 2:** Results of SNP calling by MTA-seq with 443 SNP markers and whole-genome re-sequencing.

**Accession**	**Number of obtained reads**	**Number of reads on 443 SNPs**	**Theoretical genotype**	**Number of called genotypes and its rate**							
				**AA**	**%**	**AB**	**%**	**BB**	**%**	**NC**	**%**
**MTA-SEQ WITH 443 SNP MARKERS**											
Bd21	7,503,503	3,161,811	AA	422	95.3	11	2.5	0	0.0	10	2.3
Bd1-1	7,128,438	3,628,181	BB	0	0.0	2	0.5	425	95.9	16	3.6
Bd3-1	6,109,749	2,482,031	BB	1	0.2	4	0.9	425	95.9	13	2.9
Bd21-3	5,613,548	2,194,140	BB	0	0.0	2	0.5	429	96.8	12	2.7
Bd30-1	7,095,176	3,304,472	BB	0	0.0	5	1.1	426	96.2	12	2.7
Koz-1	5,597,679	2,306,393	BB	0	0.0	4	0.9	427	96.4	12	2.7
Koz-3	5,650,458	2,326,367	BB	0	0.0	5	1.1	426	96.2	12	2.7
Koz-4	5,587,759	2,407,609	BB	0	0.0	4	0.9	432	97.5	7	1.6
Bd21 × Bd3-1	6,502,896	2,921,015	AB	6	1.4	425	95.9	4	0.9	8	1.8
**WHOLE-GENOME RE-SEQUENCING**											
Bd3-1 (DRR124340)	95,612,960	27,713	BB	1	0.2	1	0.2	440	99.3	1	0.2
Bd21-3 (DRR124339)	83,270,974	82,342	BB	0	0.0	16	3.6	427	96.4	0	0.0

### Assessment of robustness and scalability of MTA-seq

**Figure 4:**
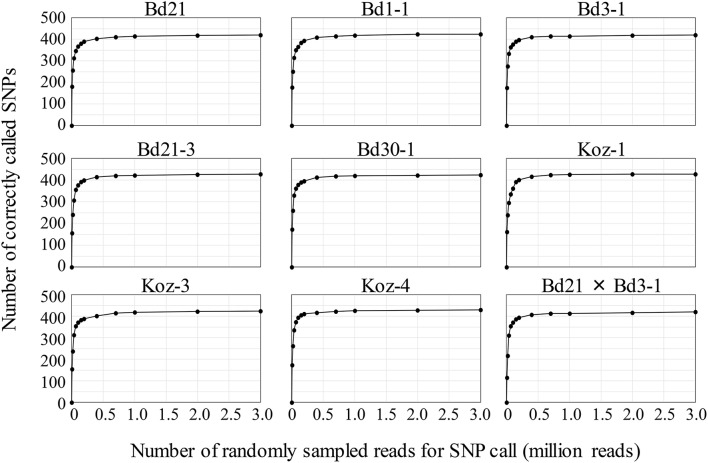
Simulation study of genotyping robustness with increasing numbers of randomly sampled reads. The 0.01, 0.02, 0.04, 0.07, 0.1, 0.15, 0.2, 0.4, 0.7, 1, 2, and 3 million NGS reads were randomly sampled from the total NGS reads obtained in this study for each accession. Data are the mean value of five independent random samplings.

To assess MTA-seq robustness and scalability, we conducted a simulation study with the 443 SNP markers designed in this study for natural accessions of *B. distachyon* and estimated the sequencing-depth threshold for accurate SNP calling. We randomly sampled 10,000 to 3,000,000 reads from the read dataset obtained in this study for each accession, followed by validation of SNP-calling accuracy for increasing numbers of sampled reads. The SNP-calling accuracy rate for an individual accession reached ~50% when using only 20,000 reads and increased to > 90% when using 400,000 reads, becoming almost saturated when using ≥700,000 (Table S4 and Figure 4). These simulation results verified the high accuracy rate of MTA-seq for a small number of sequencing reads and suggested possible scalability for genotyping of a large number of samples simultaneously.

## Discussion

In this study, we developed a high-throughput genotyping method, MTA-seq, using NGS technology and examined its reliability using 443 SNP markers designed for *B. distachyon*. Moreover, we demonstrated that MTA-seq can provide accurate genotyping data for > 400 SNP markers. MTA-seq has the following three main advantages.

### Low-quantity and/or low-quality DNA samples can be processed

MTA-seq is developed based on multiplex PCR, and, therefore, is applicable to very limited amounts of input DNA derived, for example, by laser-capture microdissection from formalin-fixed paraffin-embedded tissues (Beadling et al., 2013; Singh et al., 2013). It is also applicable to low-quantity and low-quality input DNA samples, such as those derived from dried herbarium and fossil specimens (Gugerli et al., 2005; Délye et al., 2013; Beck and Semple, 2015), which generally contain heavily degraded DNA. MTA-seq requires amplicons of only 150–200 bp for genotyping, circumventing the need for intact DNA. The genotyping of ancient DNA samples by MTA-seq will accelerate the historical study of agricultural species domestication, wild-species distributions, and plant taxa extinction with spatial and temporal resolution.

### MTA-seq is applicable to large sample numbers

For genotyping applications, such as QTL mapping and phylogeographic analysis of wild populations, methodologies that allow high-throughput processing of a large number of samples are advantageous. In this study, we adopted the Ion Xpress Barcode Adapters 1–96 kit, allowing simultaneous processing of 96 samples for library preparation. Moreover, the theoretical number of reads per run that the Ion Proton sequencer can produce is from 60 million to 80 million. Based on our study, 400,000 reads were sufficient for accurate genotyping of > 400 SNPs (90% of the 443 SNP markers) in an individual by MTA-seq (Table S4 and Figure 4). Therefore, the sequencing capacity of the Ion Proton system allows for genotyping 400 SNPs in 150–200 individuals. Our MTA-seq method is applicable to Illumina platforms, such as the HiSeq 2500 system (2 billion single reads per flow cell). When using the SPARK DNA sample prep kit for Illumina (Enzymatics), up to 5,000 individuals can be genotyped at once. NEXTflex-HT Barcodes (Bioo Scientific, Austin, TX, USA) are available for preparing up to 384 Illumina libraries, and extra barcodes for indexing up to 2,380 samples are under development. We note that MTA-seq multiplex PCR produced a remarkable number of reads (49.1–60.9% of total obtained reads) that did not map to the targeted SNP regions (Table 2). Although we did not investigate the issue in detail, this finding may be attributed to primer dimer formation or mispriming during the highly multiplexed PCR. A previous study reported that fragmented ends of sheared DNA may form incomplete primer sites, potentially resulting in the production of non-target sequences (Campbell et al., 2015). However, non-target-sequence production could be significantly improved by exonuclease I and shrimp alkaline phosphatase treatments prior to library preparation (Campbell et al., 2015). Therefore, if these problems can be solved within the MTA-seq format, this method will allow even higher throughput.

### Target SNPs can be added flexibly

One of the strongest advantages of multiplex PCR-based genotyping methods, including MTA-seq, is that they flexibly allow the addition of target SNPs of interest. For example, after QTL identification using genome-wide SNP markers as reported in this study, locus-specific SNP markers accumulated in a QTL region can easily be added to the MTA-seq format to narrow the location of the QTL and identify the genes underlying the QTL. Importantly, MTA-seq can be applied to any SNP in any organism if a reference genome is available. Conversely, a limitation of the MTA-seq method is that the DNA sequence around the target SNPs must be known in order to design primers.

### Comparison of MTA-seq with other non-commercial SNP-genotyping methods

Recently, other non-commercial SNP-genotyping methods combining NGS technology with multiplex PCR, such as a three-round multiplex PCR-based SNP genotyping method (Chen et al., 2016), and Genotyping-in-Thousands by sequencing (GT-seq) (Campbell et al., 2015), have been reported. Here, we performed a comparison between these two methods and MTA-seq with respect to factors that are important for amplicon sequencing-based genotyping.

(1) With regard to the amount of genomic DNA used as the multiplex PCR template, MTA-seq and the other two methods were found to require comparable amounts of input DNA (less than 50 ng of genomic DNA; e.g., 10–50 ng for GT-seq, 20 ng for the three-round multiplex PCR-based method, and 50 ng for MTA-seq).

(2) With regard to the size of the amplicon for genotyping, GT-seq was performed with less than 100 bp of amplicon, whereas the size of amplicon for three-round multiplex PCR-based method and MTA-seq was 107–160 and 150–200 bp, respectively. Shorter amplicon size may be more suitable for samples in which the genomic DNA is heavily degraded.

(3) The throughput and accuracy rate of MTA-seq SNP calling are comparable to, or slightly superior than, those of the two above mentioned methods. The three-round multiplex PCR-based method allowed genotyping of 37 SNPs in 757 human genomic DNA samples simultaneously, with a SNP-calling accuracy rate of 90.5%, whereas GT-seq allowed genotyping of 192 SNPs in 2,068 steelhead trout genomic DNA samples, with a SNP-calling accuracy rate of 96.4%. Using MTA-seq, we genotyped 443 SNPs in nine *B. distachyon* genomic DNA samples simultaneously, with 95.3–97.5% accuracy. Although the number of samples capable of being handled can be increased to 384 by using a greater number of adapters (e.g., NEXTflex-HT Barcodes), the relatively small sample number is the weakest point of MTA-seq, as compared with the other two methods.

(4) With respect to the length of primers, primers used in MTA-seq range from 18-31 bases in length, whereas the other two methods used much longer primers (e.g., 50–74 bp for GT-seq, and 37–38 bp for the three-round multiplex PCR-based method). The reason for the longer primer length used by these two methods is the addition of adapters by two- or three-round multiplex PCR, unlike the present MTA-seq method in which adapters are added by ligation. Generally, shorter primers are beneficial as their synthesis is relatively inexpensive.

NGS offers a powerful tool that enables users to obtain several gigabases of nucleotide sequence, with reduced cost and time. Whole-genome re-sequencing enables us to determine genetic diversity at a high resolution in an unbiased manner (James et al., 2013; Michael and VanBuren, 2015). However, several challenges arise when dealing with a large number of samples from organisms with complex and large genomes. In these cases, MTA-seq allows for high-throughput genotyping in a cost- and time-effective manner.

## Conclusion

The simple, flexible, and versatile SNP-genotyping method reported in this study (MTA-seq) will accelerate forward-genetics approaches, not only in *B. distachyon* but also in other species, including economically important temperate cereals, such as wheat and barley. The SNP marker panel for MTA-seq of *B. distachyon* and the genotype information reported in this study should be useful in various research fields, including evolutionary, ecological, agricultural, and population genetics.

## Author contributions

YO and KM: Designed the experiments and wrote the manuscript; YO and MS: Performed the experiments; YO, KT, and KI: Analyzed the NGS data; All authors read and approved the final manuscript for publication.

### Conflict of interest statement

The authors declare that the research was conducted in the absence of any commercial or financial relationships that could be construed as a potential conflict of interest.
