# Supplementary figures and images for: Multiplex PCR Targeted Amplicon Sequencing (MTA-Seq): Simple, Flexible, and Versatile SNP Genotyping by Highly Multiplexed PCR Amplicon Sequencing
Source: Front Plant Sci. 2018 Mar 23;9:201. doi: 10.3389/fpls.2018.00201 (PMC5876661; doi:10.3389/fpls.2018.00201)

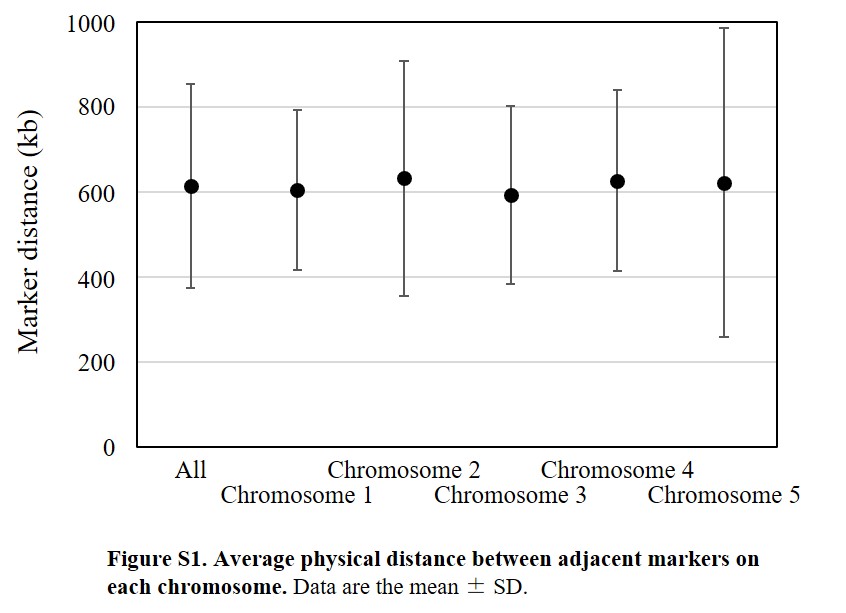

Supplement: Supplementary file 5 [file Image1.jpg]
